# A dose of realism for healthy urban policy: lessons from area-based initiatives in the UK

**DOI:** 10.1136/jech.2007.068775

**Published:** 2008-09-08

**Authors:** H Thomson

## Abstract

Many urban policies aim to improve areas and address socioeconomic deprivation. The resulting investment is often delivered through area-based programmes which incorporate initiatives to improve the physical, social and economic environment. Hypotheses that these investments can contribute to wider public health strategies are based on epidemiological data and used to support the concept of healthy urban policy. However, there is little evidence on their ability to generate positive impacts on socioeconomic or health outcomes. The lack of validating evidence on actual impacts raises two important questions: (1) Is area-based investment an effective strategy to tackle socioeconomic deprivation? (2) What is the prospect for new and improved evaluations to provide stronger evidence? Both the programmes of area investment and their accompanying evaluations have been criticised for being overly ambitious in what can be achieved by the investment and what can be measured by an evaluation. Area-based approaches to tackling deprivation have their advantages but a mix of area and individual-level targeting is likely to be needed. While there is scope to improve the utility of evaluation data there are also inevitable constraints on assessing and attributing impacts from urban investment. The inherent limitations to an area-based approach and the ongoing constraints on impact evaluation will inevitably temper expectations of what healthy urban policy can achieve. However, lack of evidence is not grounds to abandon the concept of healthy urban policy; adoption of more realistic expectations together with improved evaluation data may help to increase its credibility.

Healthy public policy, a term currently popularised by the World Health Organization, has been defined as public policy which improves living conditions; its adequacy measured by consequent health impacts.^4^ In the UK and elsewhere, there is now established political interest in using public policy as a health improvement strategy through tackling the socioeconomic roots of poor health and health inequalities.^1^ ^5^^–^10 Urban policy is a major route through which governments attempt to deliver improvements to living conditions and economic opportunities; commonly through large-scale programmes of urban regeneration or neighbourhood renewal as well as local community-based initiatives. Such investment is often area-based, targeting priority areas, and the various investment activities may be collectively described as area-based initiatives (ABIs). Area-based initiatives are, therefore, potentially central to the pursuit of healthy urban policy and more generally to healthy public policy.

Healthy urban policy is most often discussed conceptually, with little discussion of how it might be realised. This paper draws on recent efforts to exploit available evidence for healthy urban investment and points to important issues which need to be acknowledged if some form of healthy urban policy is to be pursued at an operational level. The paper reflects on the empirical support for ABIs as a strategy to tackle poor health and health inequalities, as well as broader issues of the generation and potential use of research evidence. While some of the issues raised are specific to urban policy, many are also pertinent to the broader topic of evidence-informed healthy public policy.

## ABI POLICY LINKS TO HEALTH

Area-based initiatives target geographical areas of deprivation and commonly comprise investment in key socioeconomic determinants of health, for example employment, housing, education, income and welfare. In addition to these substantive material interventions, there will often be accompanying initiatives to promote healthy lifestyles. In the UK, the official links between ABIs and health have historically been limited to specific funding themes around health initiatives, most often involving physical improvement to health service provision or promotion of healthy lifestyles—for example, smoking cessation initiatives. However, over the past 10 years the shift towards joined-up policy has led to clearer and more visible policy links between ABI investment to tackle socioeconomic deprivation and health impacts (box 1). Indeed, in the UK, neighbourhood renewal is currently an explicit part of the national strategy to tackle health inequalities.^1^ ^5^

**Box 1 The potential for health improvement is used to justify government investment in area regeneration and renewal**

- “Local neighbourhood renewal and other regeneration initiatives are in a particularly good position to address health inequalities because they have responsibility for dealing with the wider determinants that have an impact on people’s physical and mental health.”^1^
- “The benefits of including health in regeneration strategy are twofold. First there are the direct benefits of improving peoples’ physical and mental health and wellbeing. Second are the indirect benefits for employment, quality of life, levels of stress and the cost of hospital admissions or medicines.”^2^
- “Area regeneration has a key contribution to make to improving health. It tackles the social, economic, and environmental problems of multiple deprivation. And it embodies the concerted approach the government seeks to foster.”^3^

## ABI PROGRAMMES’ IMPACTS ON HEALTH AND SOCIOECONOMIC DETERMINANTS OF HEALTH: STATE OF THE EVIDENCE

There is irrefutable evidence from epidemiological research to support the hypothesis that interventions which aim to alleviate socioeconomic deprivation will lead to improved health; impact data following such interventions are needed to confirm this hypothesis. Large-scale ABIs, both in the UK and elsewhere,^11^ ^12^ have been evaluated, but much of the arising data are presented in policy reports which are often hidden in poorly catalogued grey literature and difficult to locate. Despite considerable efforts to extract what data are available, it is apparent that empirical data confirming expectations that ABIs will lead to health impacts, or other relevant socioeconomic impacts are seriously lacking.^11^^–^14 Until relatively recently the focus of much evaluation has been on audit; reporting monies spent and gross outputs, such as miles of new road built, or number of training places provided, rather than actual impacts—that is, changes over time compared with baseline. The growing interest in assessment of impacts is illustrated by the emergence and the gradual improvement of impact evaluations over the past 15 years.^11^ ^15^ ^16^

The data generated by the evaluations of national UK ABI programmes have been reviewed to produce an evidence synthesis,^11^ (table 1) albeit limited by the quality, quantity and nature of the data available. Health and mortality impacts have been assessed in four evaluations, but conflicting data make it impossible to draw conclusions about the health impacts of previous ABIs. Employment and education impacts are the most commonly reported socioeconomic impact; data are suggestive of a modest positive impact. Impacts on income and housing quality have rarely been assessed, making it difficult to generalise about likely impacts.^11^ Notably, the ongoing evaluation of the New Deal for Communities (NDC) ABI programme, includes a panel survey following the same individuals in both NDC areas and a sample from similarly deprived neighbourhoods within the same geographical area but which are not part of the NDC programme.^15^ ^16^

**Table 1:** Summary of evidence of impacts on health and socioeconomic determinants of health from national programmes of urban regeneration in the UK (1980–2004)

Health impacts	Rarely assessed - conflicting findings (5 evaluations)
Employment	Modest positive impact (10 evaluations)
Education	Modest positive impact (6 evaluations)
Housing quality	Rarely assessed (1 evaluation)
Income	Rarely assessed (3 evaluations)

From the scant amount of impact data available there is much uncertainty around whether ABIs do impact positively on health or the socioeconomic determinants of health; with even less known about the social distribution of impacts and the implications for health inequalities. It is important to remember that this uncertainty should be interpreted as absence of evidence rather than evidence of absence.

The lack of evidence and uncertainty about impacts raises two fundamental issues which need to be addressed if ABIs are to be incorporated into a strategy to improve health and reduce health inequalities. Firstly, there is the question of whether area-based approaches can be effective at targeting socioeconomic deprivation; the use of ABIs as a strategy for health improvement assumes they are. And secondly, to what extent and how can evaluations be improved to provide a stronger evidence resource with which to improve the effectiveness of future urban policy (whether or not as part of a wider health improvement strategy)?

## ARE ABIS AN EFFECTIVE STRATEGY TO TACKLE SOCIOECONOMIC DEPRIVATION?

Area-based initiatives and their approach to targeting small areas are an efficient way to deliver intensive periods of investment to a target population concentrated in a small area, and may also alleviate negative area effects that may be associated with concentrations of multiple deprivations in a small area. There may also be added value in terms of the local agency synergy and partnership when concentrating investment in a small defined area.^17^

However, there have also been some serious criticisms of ABIs and their approach of targeting small areas. At a policy level, ABIs have been criticised for being short-term, unfocused, and overly ambitious given relatively modest funding.^18^^–^22 The continually changing political landscape, local, national and global, inevitably limits the potential impact of any single policy, including relatively short-term ABI investment.^19^ A further related criticism is that predictions of positive impacts are made with no clear underlying theory of what type of impacts and how such impacts will be achieved. The diverse range of interventions delivered by ABI programmes, ranging from rehousing, employment initiatives and environmental improvements, to healthy lifestyle initiatives, means that the types of and routes to possible health outcomes and socioeconomic outcomes will be diverse. Some of these interventions may have a direct, observable impact which does not need further elaboration or exploration, for example impact of graffiti removal on neighbourhood aesthetics. However, immediate observable impacts cannot always be relied upon and expectations that the investment will lead to impacts, health or socioeconomic, would benefit from a more explicit theory mapping the types of impacts expected, timeframes, affected populations, and mechanisms for impacts.

The use of an area-based approach to target deprivation is also problematic. Although there are well-defined areas with concentrations of multiple deprivations, it has been repeatedly demonstrated that, in the UK at least, the majority of socioeconomically deprived individuals do not live in these areas. Thus, ABIs have been criticised for missing the majority of the target population.^17^ ^23^ Area-based approaches have also been linked to possible stigmatising of an area.^24^ ^25^ Identifying a local area as an ABI area publicly labels the area as deprived and may add further to social exclusion of the area and its residents.^24^^–^27 The use of an area-based approach has also been dismissed as being an inadequate sticking plaster to address the roots of socioeconomic deprivation and social exclusion, which do not necessarily stem from the area itself but rather are more deeply rooted at a societal level.^20^ ^28^

Aside from research evidence, other, more casual, observations have noted that despite decades of ABI investment, deprivation persists in many target areas; this brings into question the effectiveness of ABIs.^18^ ^29^ A counterargument in support of ABIs is that residents of ABI areas, known to be highly mobile, who are benefiting from improved socioeconomic circumstances, often leave the area and are replaced by individuals experiencing higher levels of socioeconomic deprivation.^17^ ^19^ Thus, although individuals are benefiting, the investment may be appearing to fail as the target area remains a ghetto for the most socially excluded.

The above criticisms suggest that expectations of significant socioeconomic impacts following ABI investment may be unrealistic. How much more unrealistic, therefore, is the expectation of substantial health impacts, which is predicated on the effectiveness of ABIs to impact on socioeconomic outcomes?

The alternative to targeting small areas is to target individuals in large areas, nationally or regionally. Such an approach is more likely to reach the majority of the socioeconomically deprived population who will not be reached by ABIs targeting the most deprived small areas. In addition, large-area targeting is more likely to be part of existing mainstream funding rather than short-term grants, and may be less likely to lead to stigmatisation of an area. Inevitably, there are pros and cons to both approaches^17^ ^28^, and appropriate mix targeting small areas and individuals would appear to be desirable.^17^ ^19^ There is no doubt about the relative merits of targeting small areas, and the criticisms levied at ABIs do not justify abandoning targeting small areas, but rather it is important to be aware of the strengths and limitations when prospectively assessing the potential impacts.

## NEW IMPROVED EVALUATIONS: THE ANSWER?

It is well established that little is known about the impacts of ABIs. This dearth of evidence would appear to be largely due to a lack of research, suggesting that there is potential for new primary studies to address this knowledge gap. The past decade has witnessed calls for more evidence to support public policy generally through the use of new and improved impact evaluations.^30^ ^31^ In particular, there have been calls for evaluations that use quasi-experimental designs.^32^ ^33^

An examination of previous evaluations in this field points to some obvious areas that need to be improved. Like the programmes themselves, ABI evaluations have been criticised for being over-ambitious in what they expect to be able to assess within the set time and resource constraints.^18^ ^19^ ^34^ This is especially relevant where impacts of interest, such as health, cannot be expected in the short term. Other criticisms of previous ABI evaluation include the absence of a theory-based approach to test hypothesised mechanisms for the key impacts being assessed;^11^ ^12^ ^18^ ^19^ ^34^ ^35^ an over-reliance on routine data rather than cohort studies to track individuals;^11^ and the lack of comparison data to give an indication of additionality (ie what impacts have occurred in addition to those that would have occurred regardless of the investment).^11^ ^34^ Moreover, much of the data presented in available evaluations is incomplete and difficult to interpret as the description of methods used is often unclear.^11^ ^36^

Some of the above criticisms can be addressed simply and without much cost; though may require more careful thought in evaluation design. In line with the need for the links between intervention and predicted impacts to be supported by more explicit theory or pathways, evaluations should be designed to test these theories (using their own theory where none have been previously devised).^35^ Other ways in which the utility of evaluation data could also be increased relatively inexpensively include improved clarity of reporting of results and methods, and substituting assessments of distal health outcomes with proxy measures of health determinants, using either routine or self-reported measures.^8^ Innovative use of routine data has been recommended as a practical, low-cost resource for evaluation.^37^ For example, where routine data are available for small areas, it may be possible to carry out a time-series analysis, comparing projected trends from before the intervention with actual trends observed following the investment.^38^ Area-based routine data are unable to report changes among individuals, and this presents an obstacle given the typical mobility of residents in ABI areas.^17^ ^19^ However, recent improvements in the availability of small-area data more closely reflect the defined target area or neighbourhood and should provide increased utility of routine data. For example, in the UK, useable routine data on numerous socioeconomic outcomes are now available,^39^ and in Scotland much of this is available for small areas of around 750 people.^40^ In addition, linking routine individual health service data to individual neighbourhood survey data is now possible.^41^ Where routine data are not available for geographies that relate closely enough to the intervention area, there may be no worthwhile alternative to intensive and costly panel surveys of individual residents.

In addition to quantitative assessments, qualitative data can shed light on unforeseen impacts, and can also provide valuable insights into possible pathways for impacts. Assessments from both those delivering and those in receipt of the intervention may provide helpful contrasts in perceptions of the intervention and its impacts, and may also explain unexpected impacts or the distribution of impacts.

**Box 2 A summary of key issues to consider in the realistic pursuit of healthy urban policy**

**Realistic aims:** Are the expected impacts and timescales realistic given the level of funding and timescale of the programme and the evaluation?

**Why area-based investment might not be as successful as hoped**

**Area versus individual investment:** Investment targeted at priority areas will not reach the majority of socioeconomically deprived population at a national level.

**Theory:** Mechanisms or routes through which impacts are to be expected need to be made explicit when the programme is being planned (this is aside from visible links to health within the general vision of a programme). This has rarely been done and programmes have been criticised for being unfocused.

**Residential mobility:** Residents whose socioeconomic circumstances improve often leave the area and are replaced by more socioeconomically deprived residents, thus area-based deprivation remains despite apparent benefit for some individuals.

**Stigma:** Areas in receipt of assistance may be stigmatised, thus compounding existing social exclusion for residents.

**Ideological:** Area-based initiatives have been criticised for ignoring the root causes of socioeconomic deprivation and exclusion at wider societal level.

**Issues when assessing the health impact of area-based investment**

**Use of theory:** Evaluations should be designed to test a pre-specified theory mapping a mechanism or route to a measurable outcome (see above).

**Reporting of data/methods:** Improved transparency of evaluation methods and reported results would improve the utility of evaluation data.

**Individual or routine data:** Routine data is inexpensive but is often limited in reporting changes at individual level.

**Small effect size:** Detecting small health effects will require a large study population to detect significant changes at a population level.

**Recruitment of target population:** Response rates in areas of deprivation are falling.

**Comparison areas:** Use of a suitable comparison area is desirable but identification of an area with equal need but not selected for the investment is difficult.

**Defining exposure to intervention:** Individuals within the target area will have widely varying levels of exposure to what are often multiple interventions.

**Time-scale:** Timing of final outcome is unknown but may be many years after the intervention. Aside from resource implications, and attrition, long-term follow-up may have an effect itself, and introduces additional confounding due to the passage of time. An alternative is to use proxy measures which can be measured within 2–3 years, eg socioeconomic determinants of health.

**Defining success:** Slowing the rate of downward trends may be an important success, but this may be wrongly reported as a negative impact; assessing trends before and after the intervention may be required.

Other criticisms of ABI evaluations may be more difficult to address. Conducting community based, quasi-experimental evaluations, which are powered to detect small impacts among individuals over long periods is neither straightforward from a pragmatic point of view,^42^ ^43^ nor cheap. Area-based initiatives comprise multiple and varied interventions delivered over a period of time. Typically, it will not be possible for the evaluators to control the allocation or timing of the intervention. Details of the nature, implementation and timing of the interventions can be invaluable to the evaluation but obtaining this information requires time and skills to develop good relationships with key stakeholders. Furthermore, there is the increasing problem of high levels of attrition in deprived areas, which are most likely to be targeted by ABIs.^44^

Aside from the considerable cost implications and difficulties of implementing a rigorous evaluation in the ‘real world’, even an evaluation which achieves 100% response and follow-up levels at 10 years or longer may well still fail to generate the hoped-for evidence due to the introduction of confounding factors. Even in the short term, impacts are likely to occur in conjunction with other changes which may or may not be associated with the intervention. Extended follow-up inevitably introduces further multiple confounding due to other changes over time, be they at an individual, area or societal level; and intensive, longitudinal studies tracking individuals may themselves need to be quite interventionist and, thus, introduce an additional confounder which is difficult to control for.

Other conceptual problems for these evaluations include definitions of exposure and success, and identifying comparison areas. Area-based initiatives involve multiple interventions, ranging from rehousing, environmental transformation to health promotion initiatives. The different components of the interventions typically target relevant subgroups within the investment area. Yet categories of exposure to the intervention are often reduced to a binary variable which is insensitive to the varying exposures experienced within the target population and its sub-groups. Similarly, any number of diverse outcomes may be used to assess the impact of the various interventions and may well report a mix of effects, making an overall assessment of success difficult.

Definitions of success may refer to positive impacts for the target area but this alone is unable to reveal whether or not the impacts would have occurred, and may even have been greater, had the area not received any intervention. Conversely, a negative impact cannot be assumed to indicate failure; without the investment the area may have fared worse. Analysis of trend data and comparison area data can help illuminate additional change, but identifying areas which are similar sociodemographically at a detailed level, as well as being in equal need of the ABI investment, may not be possible.^45^

**What is already known on this subject**

The concept of healthy urban policy is intuitively appealing to both urban policy makers and the public health community. But area-based initiatives and their attendant evaluations are often over-ambitious relative to the funding levels and timescales allowed. With little prospect of obtaining clear empirical support, the pursuit of evidence informed healthy urban policy may be dismissed as idealistic.

**What this study adds**

- Despite the difficulties in gathering evidence of the impacts of area-based investment, the overwhelming epidemiological evidence supporting the hypothesis that alleviating socioeconomic deprivation will generate health improvement suggests that healthy urban policy is still worth pursuing.
- Policy makers and evaluators need to agree realistic expectations of how urban policy might impact on health and its determinants, and what evidence is obtainable within the resource and conceptual limitations of an evaluation.

## CONCLUSION

With little prospect of robust empirical validation, the development of evidence-informed healthy urban policy may be dismissed as an impossible ideal; but this is not grounds for total abandonment of the concept. Aside from health improvement, investment to alleviate socioeconomic deprivation can be justified on grounds of social justice. Support for the concept of healthy urban policy and forecasts of accompanying health improvement relies on the well-established links between socioeconomic deprivation and health;^46^ data from outcome evaluations are needed to validate these forecasts, but is currently lacking and may be difficult to obtain.

The continued pursuit of healthy urban policy needs to incorporate a more realistic and pragmatic approach (box 2). Policy makers and evaluators need to set agreed expectations of both area-based investment and its evaluation. This requires a clear acknowledgement of the inevitable uncertainties, while also incorporating scope for improvement using empirical evidence from evaluations. Some practical solutions, discussed earlier, could greatly increase the utility of evaluation data, but expectations still need to be tempered by what evaluation can realistically achieve. In time, evaluation of realistic (both achievable and measurable) impacts should provide ‘best available’ evidence to inform how best to mitigate possible harms and maximise benefits of future urban investment.
